# Supplementary material for: Content-rich biological network constructed by mining PubMed abstracts
Source: BMC Bioinformatics. 2004 Oct 8;5:147. doi: 10.1186/1471-2105-5-147 (PMC528731; doi:10.1186/1471-2105-5-147)
Supplement: Additional File 2 — The original results of the above study (non-essential files are deleted to keep the file size under the limit set by BMC bioinformatics). [file 1471-2105-5-147-S2.bz2 › chilibotAdditionalFile2/dip20/582ID9571026E10950/html/GHR_GH1.html]

 


 **GHR** and **GH1** 
  
Found 60 abstracts in PubMed, retrieved 20.  
 

 What does Google say? 
 PDF only 
| .edu only 

---

**Interactive relationship** (e.g. stimulation, inhibition, etc)

**Non-interactive relationship** (e.g. studied together, co-existance, homology, etc.)

- Studies have shown that bovine placental lactogen bPL has partial somatogenic activity in vivo even though binding results clearly indicate bPL does not cause homodimerization of the bovine somatotropin  [ **GH1** ]  receptor  [ **GHR** ]  bST R.  Ref: 10622285 Life Sci, 1999
- Expression of growth hormone receptor  [ **GHR** ]  1A messenger ribonucleic acid in liver of dairy cows during lactation and after administration of recombinant bovine somatotropin  [ **GH1** ] .  Ref: 10509249 J Dairy Sci, 1999
- Pituitary growth hormone  [ **GH1** ]  GH is essential for postnatal growth in animal, it regulates numerous cellular functions by direct effect on it s receptor  **GHr**  in many different tissues.  Ref: 12096625 Yi Chuan Xue Bao, 2002
- The objective was to characterize P1 activity by measuring the amount of growth hormone receptor  [ **GHR** ]  1A mRNA in liver of dairy cows at different stages of lactation as well as after administration of recombinant bovine somatotropin  [ **GH1** ]  rbST.  Ref: 10509249 J Dairy Sci, 1999
- We therefore determined the expression of growth hormone GH receptor  [ **GHR** ]   **GHR** , and insulin like growth factors I and II IGF I and IGF II mRNA in liver and skeletal muscle longissimus of neonatal pigs given daily intramuscular injections of either recombinant porcine GH 1  [ **GH1** ]  mg kg body wt.  Ref: 10749770 Am J Physiol Regul Integr Comp Physiol, 2000
- Evaluation of the somatogenic activity of bovine placental lactogen with cell lines transfected with the bovine somatotropin  [ **GH1** ]  receptor  [ **GHR** ] .  Ref: 10622285 Life Sci, 1999
